# Novel Methods for Personalized Gait Assistance: Three-Dimensional Trajectory Prediction Based on Regression and LSTM Models

**DOI:** 10.3390/biomimetics9060352

**Published:** 2024-06-12

**Authors:** Pablo Romero-Sorozábal, Gabriel Delgado-Oleas, Annemarie F. Laudanski, Álvaro Gutiérrez, Eduardo Rocon

**Affiliations:** 1 BioRobotics, Centro de Automática y Robótica, Consejo Superior de Investigaciones Científicas–Universidad Politécnica de Madrid (CSIC-UPM), 28500 Madrid, Spain; pablo.romero@csic.es (P.R.-S.); gabriel.delgado@csic.es (G.D.-O.); e.rocon@csic.es (E.R.); 2 Ingeniería Electrónica, Universidad del Azuay, Cuenca 010107, Ecuador; 3 Faculties of Engineering and Medicine, School of Biomedical Engineering, Dalhousie University, Halifax, NS B3H 4R2, Canada; annemarie.laudanski@dal.ca; 4 ETSI Telecomunicación, Universidad Politécnica de Madrid, 28040 Madrid, Spain

**Keywords:** gait pattern, regression, LSTM, robotic gait support

## Abstract

Enhancing human–robot interaction has been a primary focus in robotic gait assistance, with a thorough understanding of human motion being crucial for personalizing gait assistance. Traditional gait trajectory references from Clinical Gait Analysis (CGA) face limitations due to their inability to account for individual variability. Recent advancements in gait pattern generators, integrating regression models and Artificial Neural Network (ANN) techniques, have aimed at providing more personalized and dynamically adaptable solutions. This article introduces a novel approach that expands regression and ANN applications beyond mere angular estimations to include three-dimensional spatial predictions. Unlike previous methods, our approach provides comprehensive spatial trajectories for hip, knee and ankle tailored to individual kinematics, significantly enhancing end-effector rehabilitation robotic devices. Our models achieve state-of-the-art accuracy: overall RMSE of 13.40 mm and a correlation coefficient of 0.92 for the regression model, and RMSE of 12.57 mm and a correlation of 0.99 for the Long Short-Term Memory (LSTM) model. These advancements underscore the potential of these models to offer more personalized gait trajectory assistance, improving human–robot interactions.

## 1. Introduction

Since the development of robotic gait devices for support and rehabilitation began, enhancing human–robot interaction to offer more tailored solutions has been a primary area of research focus [1]. This evolution is driven by the need to enhance embodiment and improve potential outcomes for assistance/rehabilitation tasks. To this end, a better understanding of human motion is required to successfully personalize the gait assistance provided by such devices.

Traditionally, references for gait trajectories in robotic assistance devices have been determined through pre-set patterns based on Clinical Gait Analysis (CGA) [2]. While these methods have historically been widely applied as gait pattern reference trajectories across multiple robotic platforms [3,4,5], they are not without limitations. Specifically, such methods rely on predetermined trajectories that do not account for inter-individual gait variability due to morphological differences or gait condition variability, for example, walking speed or step length, parameters that provide a rigorous basis for estimating kinematic profiles [6].

Recently, however, the development of gait pattern generators has advanced significantly to address the variability in human gait and provide more personalized assistance solutions [7,8,9]. Initially, these advancements involved the implementation of regression-based models, which have been extensively applied to analyse measured gait kinematics across diverse conditions and multiple subjects. Yun et al. [10] explored angular gait pattern estimation using Gaussian Process Regressions (GPR), correlating 14 body parameters to joint motion in 113 subjects. Koopman et al. [11] utilized regression models to reconstruct the gait of 15 participants by dimensionally reducing their gait cycles to key-events. Most recently, Hu et al. [12] examined the use of Least Absolute Shrinkage and Selection Operator (LASSO) regression models to estimate Fourier coefficients that reconstruct lower-limb angle trajectories. While these models are invaluable for establishing a baseline understanding of gait kinematics and their variability under consistent conditions, they inherently lack the flexibility needed to adapt to transient changes within gait cycles.

To complement regression models and enhance adaptability towards transient gait variability, recent studies have explored the integration of Artificial Neural Network (ANN) techniques specializing in time-series forecasting for gait pattern estimation [13,14,15,16,17]. Luu et al. [14] evaluated a dimensionality reduction method that, when combined with Generalized Regression Neural Networks (GRNN), was applied to 70 adults walking at slow and normal velocities to predict sagittal plane angular trajectories for the hip and knee. Wu et al. [18] used Gaussian regression-based models to map relationships between body parameters and gait features, reconstructing gait trajectories using encoder-decoder neural network approaches for joint angle estimations in the sagittal plane. Further research has investigated specialized Recurrent Neural Networks (RNNs), including Gated Recurrent Units, to predict angular gait trajectories from 137 subjects [19]. Zaroug et al. [15] assessed the performance of another RNN type known as Long Short-Term Memory network, training the model with six participants who walked at a consistent speed (5 km/h) using a sliding window approach to forecast the linear accelerations and angular velocities of the thigh and shank, achieving a correlation of 0.98 with the testing set. Su et al. [17] explored Long Short-Term Memory (LSTM)-based models for predicting gait phases and trajectories, achieving similar accuracy in predictions of angular velocities 100–200 ms in advance for thigh, shank, and foot segments using IMU data from 12 subjects walking at five different speeds. Additionally, Haneul et al. [20] examined gait phase recognition using a bi-directional LSTM, utilizing IMU data from sensors on the shank and feet of three subjects, achieving an accuracy of 86.43%. This growing interest in the development of more accurate gait pattern generators recognizes the need for tools which can provide not only robust baseline estimates of gait patterns but also dynamically adapt to the changing conditions of the individual during walking.

In the broader context of robotic gait rehabilitation platforms, both regression and ANN-based models play crucial roles in generating reference trajectories. Regression models are invaluable for creating stable and reliable trajectories, especially in scenarios involving patients with severely affected gait patterns or when precise historical gait data is lacking. These models are particularly effective as they ensure rehabilitation can progress effectively, even in the absence of reliable patient-specific data. Conversely, ANN models excel at handling real-time variability, which makes them ideal for fine-tuning adaptations to match the specific kinematics of healthy or less affected individuals whose movements are consistently reliable. However, ANNs face the significant challenge of the ‘black box’ phenomenon, where the decision-making processes remain opaque. Additionally, LSTM models, a type of ANN, require substantial computational resources, making them less feasible for real-time applications on hardware with limited processing power. This limitation is particularly problematic for very affected subjects whose unreliable kinematics demand high accuracy and safety in rehabilitation. The opacity of ANNs thus poses a considerable hurdle in clinical applications that require high levels of transparency and accountability.

However, one notable limitation persists across the aforementioned gait pattern generators, as they solely focus on estimating angular-space trajectories. While effective within their scope, these models fall short when applied to end-effector robotic systems, which require precise control over the three-dimensional spatial positioning of joints. Although there are some works related to three-dimensional pose estimation of human joints, these are primarily oriented toward applications that do not necessitate the clinical precision required for gait rehabilitation, such as pedestrian pose estimation for autonomous system safety, surveillance, and security during human–robot collaboration [21,22,23]. Few works have successfully bridged the gap between angular gait generators for gait rehabilitation purposes and comprehensive three-dimensional spatial trajectory predictions [24,25].

In response to this limitation and literature gap, we here introduce a novel approach that expands the state-of-the-art application of regression and ANN models beyond angular estimations to include precise three-dimensional spatial predictions. Specifically, our work studies multivariable regression techniques and LSTM models to provide dynamic and three-dimensional gait pattern predictions for end-effector gait assistance devices. We first present a multivariable regression model designed to handle the three-dimensional variability of human motion across the lower joints (hip, knee and ankle). This model utilizes the kinematic data from 42 individuals walking at eight speeds. The kinematic data were reduced to key-points which could then be used to train robust multivariable regression models for the reconstruction of gait trajectories. Complementing the regression model, we next present the integration of an LSTM network designed to predict one-step-ahead gait trajectories, corresponding with the future 100 time-normalized points given data from the previously measured 100 points.

## 2. Materials and Methods

### 2.1. Gait Dataset Description

This study utilized a publicly available gait dataset [26], which consists of 42 healthy individuals categorized into two cohorts: 24 young adults (mean age 27.6 years, height 171.1 cm, weight 68.4 kg) and 18 older adults (mean age 62.7 years, height 161.8 cm, weight 66.9 kg); detailed demographic and physical characteristics are presented in Table 1. Each participant was instrumented with 28 markers on the pelvic and lower-extremity segments, adhering to the skin-mounted marker protocol by Leardini et al. [27]. Joint trajectories were captured using a 12-camera high-speed motion-capture system at 150 Hz (Raptor-4, Motion Analysis Corporation, Santa Rosa, CA, USA) while ground reaction forces were measured using a dual-belt instrumented treadmill at 300 Hz (FIT, Bertec, Columbus, OH, USA).

**Figure 1:**
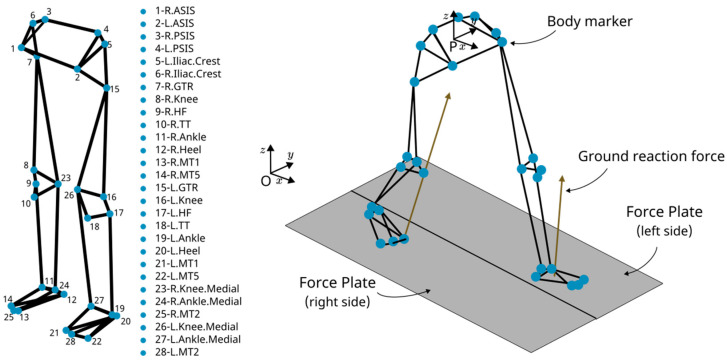
Experimental setup diagram illustrating the location of markers on the subject’s body and force plates positions.

For each participant session, a total of eight trials were recorded. During these trials, participants were asked to walk at eight gait speeds, derived from their self-selected comfortable walking speed using the Froude number [28] as follows: 40%, 55%, 70%, 85%, 100%, 115%, 130%, and 145%. Each walking trial was recorded for 90 s, wherein data from the final 30 s was analysed as stationary walking. Visual overviews of these conditions are provided in Figure 1.

### 2.2. Gait Analysis

Marker positions and ground-reaction forces were processed using MATLAB© version 2020b to derive joint kinematics and segment the trajectories into time-normalized gait cycles. Initially, joint positions were calculated relative to the motion capture system’s origin (O). To isolate the joint movements relative to the body and accommodate for treadmill motion, these positions were then adjusted to reflect their placement relative to the subject’s pelvis (P), see Figure 1. This correction ensures the joint trajectory data accurately represents the actual movement of the body, independent of the treadmill’s influence.

**Figure 2:**
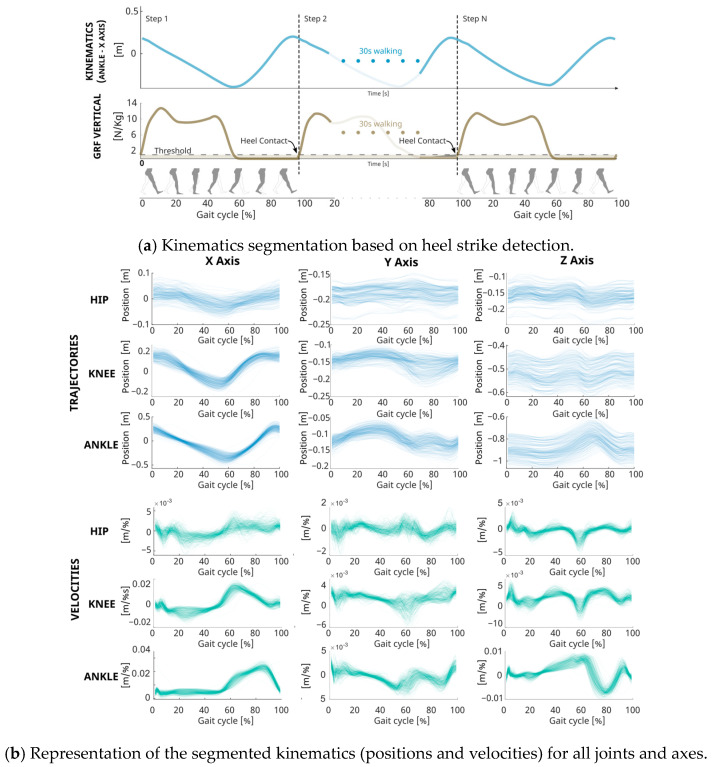
Comprehensive Gait Analysis Methodology and Kinematic Data Visualization. (**a**) Data Segmentation graph depicting how gait cycles are delineated using heel strike events detected by ground reaction forces on the force plate, with step transitions highlighted over time. (**b**) Segmented kinematics displaying the trajectory of joint positions and velocities compiled from all trials across the subject cohort.

Gait cycle normalization involved defining the start and end of each cycle using heel contact, identified from vertical force plate reactions when exceeding a threshold, see Figure 2a. The trajectories of both legs were segmented at these points to mark 0% and 100% of the gait cycle. Steps whose time duration deviated significantly from the norm, falling outside the 25–75% interquartile range of cycle duration of the trial, were excluded as outliers to ensure data integrity. This process resulted in a dataset comprising 15,531 steps, each carefully vetted for consistency and accuracy in representing gait dynamics. For a detailed visualization of these segmented trajectories, refer to Figure 2b.

### 2.3. Regression-Based Gait Pattern Generator

Human gait represents a complex sequence of movements, intricately coordinated and varying significantly among individuals due to unique physical conditions and differences in walking speeds [8]. In response to this diversity and the requirement for personalized gait analysis, our approach uses multivariable regression methods to reconstruct the three-dimensional gait trajectories.

#### 2.3.1. Gait Key-Points

To manage the inherent complexity in gait data, we adopted a down-sampling strategy similar to those presented in [11,24]. Initially, for each subject, all steps within a walking trial were averaged, resulting in nine averaged trajectories per subject’s session, corresponding to the X, Y, and Z axes of the hip, knee, and ankle of both legs. Subsequently, various key-points (*k*) representing the positions and velocities of each joint along each of their axes were extracted from the averaged trial data. Each key-point is parametrized as k(t,y), with *t* representing the percentage of the gait cycle at which the key-point occurred, and *y* representing the position in the corresponding axis. 

**Figure 3:**
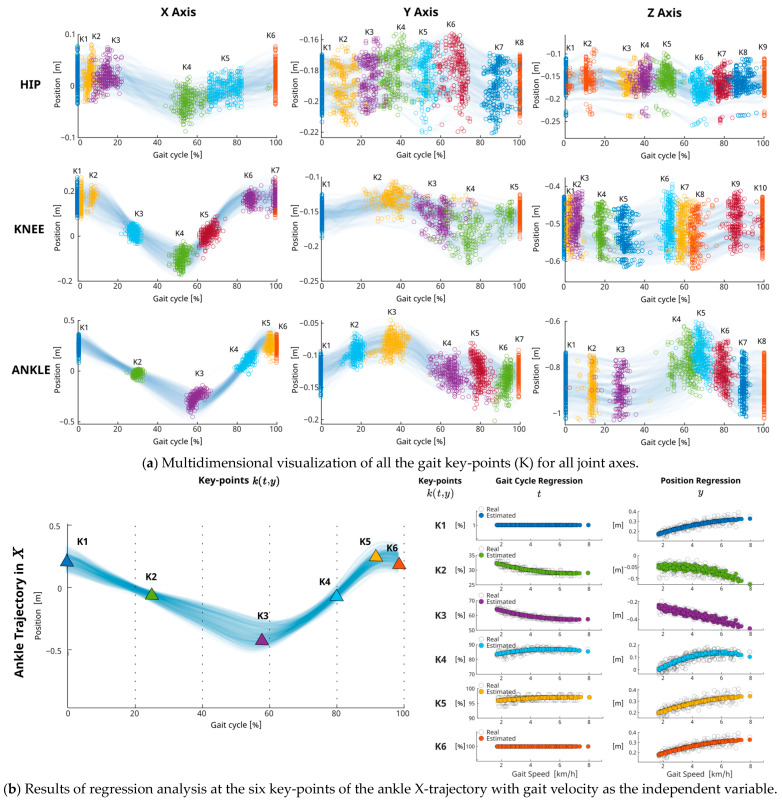
Multidimensional analysis of gait key-points along all joints and axes and example of regression-based key-points estimations: (**a**) the spread of key-points for the hip, knee, and ankle joints across the X, Y, and Z throughout various gait cycles and velocities for the entire subject group; (**b**) example of key-point estimation for the ankle trajectory in X, correlating joint position and gait cycle percentage of each key-point with gait speed through regression-based analysis.

The number of key-points selected for each joint axis varied according to the complexity of the trajectory, with a total of 66 key-points retained, as detailed in Table A1, Table A2 and Table A3 and in Figure 3a. Selection was based on an analytical process assessing the contribution of each point to the overall gait pattern shape. A stepwise regression was conducted to evaluate the selected points and verify their influence on characterizing gait velocity and height. Statistical significance was evaluated at an alpha level of 0.05.

#### 2.3.2. Regression Analysis on Key-Points

To estimate the characteristic three-dimensional trajectories, robust regressions [29] with bi-square weighting functions were applied to predict the parameter values (*y*, *t*) of the previously selected key-points (*k*). The regression formula employed was as follows:

(1) $$Y=\beta_{0}+\beta_{1}\;v+\beta_{2\;}v^{2}+\beta_{3\;}l,$$

where $\beta_{i}$ are regression coefficients with i in [1,3], l represents the subject height, and v represents gait velocity. The squared v term was included in this equation to capture the non-linearities of joint kinematics [9].

This approach aims to pinpoint characteristic parameters from within the key events that enable the prediction of joint trajectories, taking into account participant-specific conditions such as height and gait velocity, Figure 3b.

#### 2.3.3. Trajectory Reconstruction

**Figure 4:**
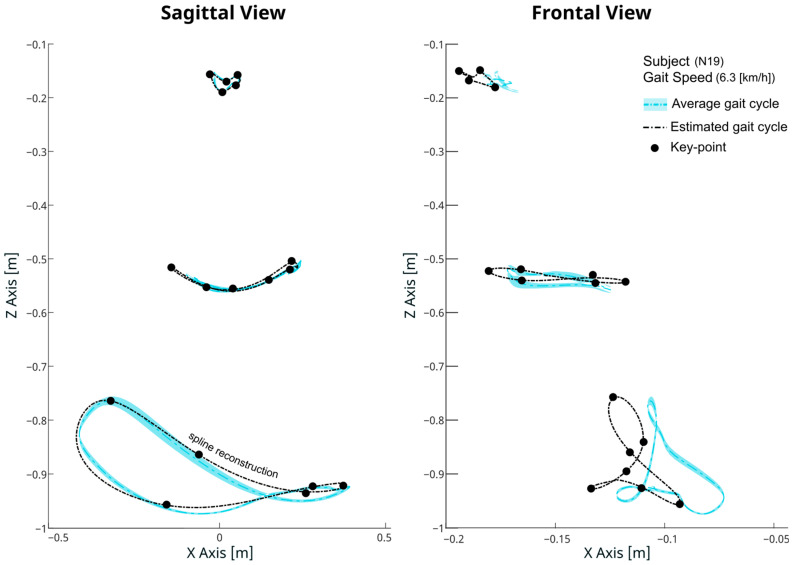
Comparative visualization of measured and reconstructed gait trajectory for a representative subject. The original, measured 3D joint trajectories (solid lines) are presented alongside the estimated trajectories (dashed lines) generated through regression-based models. The spline reconstruction method is applied to the estimated key-points (displayed in black), demonstrating the smooth and close approximation of the reconstructed path to the measured trajectory.

To obtain the final three-dimensional trajectories, spline interpolations are used to join the estimated key-points and reconstructed trajectories, ensuring a smooth and continuous linkage of key-points, thereby accurately recreating the entire gait cycle as illustrated in Figure 4.

### 2.4. LSTM-Based Gait Pattern Generator

Long Short-Term Memory (LSTM) networks are a specialized subtype of Recurrent Neural Networks that excel in time-series analysis due to their specific architecture, designed to address the challenges of their long-term dependencies [30]. LSTMs incorporate a sophisticated cell structure with multiple gates that manage the flow of information, allowing the network to retain or discard data dynamically, see Equations (2)–(7). In the context of gait analysis, LSTMs offer potential advantages due to their ability to capture temporal dependencies and variability in gait patterns.

(2) $$f_{t}=\sigma \left(W_{f}\cdot \left[h_{t-1},x_{t}\right]+b_{f}\right)$$

(3) $$i_{t}=\sigma \left(W_{i}\cdot \left[h_{t-1},x_{t}\right]+b_{i}\right)$$

(4) $$\tilde{C_{t}}=\tanh\left(W_{C}\cdot \left[h_{t-1},x_{t}\right]+b_{C}\right)$$

(5) $$C_{t}=f_{t}*C_{t-1}+i_{t}*\tilde{C_{t}}$$

(6) $$o_{t}=\sigma \left(W_{o}\cdot \left[h_{t-1},x_{t}\right]+b_{o}\right)$$

(7) $$h_{t}=o_{t}*\tanh\left(C_{t}\right)$$

where $f_{t}$ is the forget gate at time t, σ is the sigmoid function, W represents the weight matrices of the gates, $h_{t-1}$ is the previous hidden state, $x_{t}$ is the current output, $b_{x}$ are the biases of the gaits, $i_{t}$ is the input gate, $\tilde{C_{t}}$ is the candidate for addition to the cell state, $C_{t}$ is the new cell state at time t, $o_{t}$ is the output gate’s activation at time t and $h_{t}$ is the hidden state at time t.

#### 2.4.1. Data Preparation and Processing 

For LSTM analysis, the 3D kinematic trajectories obtained from the aforementioned gait dataset (previously described in Section 2.1) were utilized to train, validate and test the model. Data were normalized based on the maximum and minimum values of each joint axis, height, and gait speed. To facilitate the LSTM network’s performance, the dataset was strategically partitioned by assigning 60% of the walking sessions to the training set, 10% to the validation set, and the remaining 30% to the testing set, ensuring no overlap in participants between these sets.

#### 2.4.2. LSTM Model Architecture 

**Figure 5:**
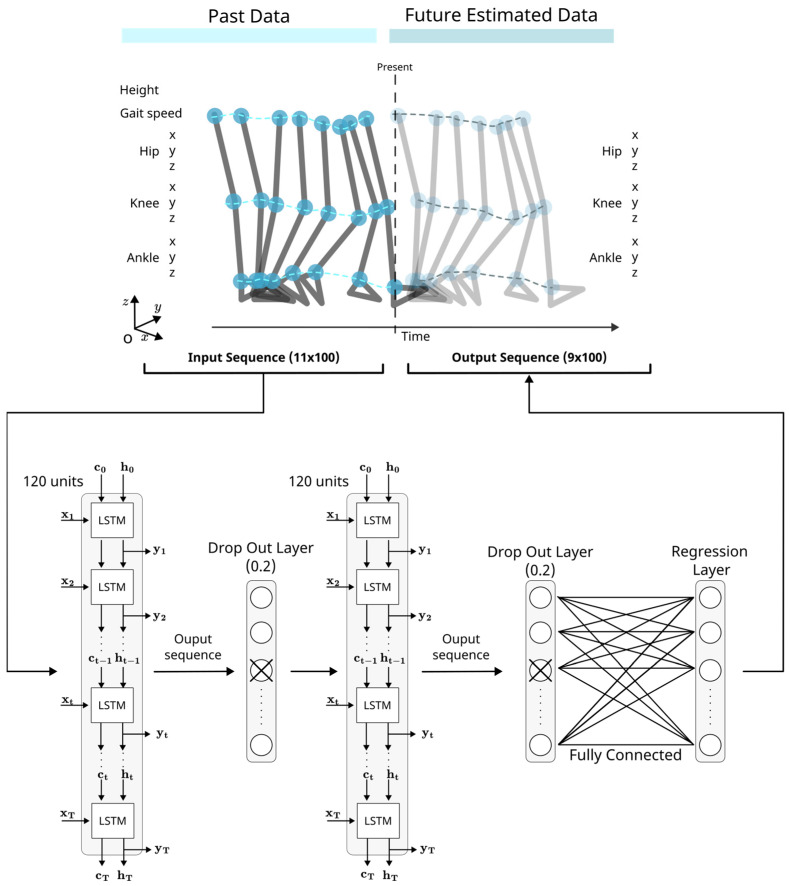
Schematic of the LSTM Neural Network to predict 3D positions of the lower joints one step ahead. Sequences of 11 features are received by the input layer, including height, gait velocity, and the positions of all lower extremity joints in the space. The network comprises two LSTM layers, each with 120 hidden units connected by dropout layers with a rate of 0.2 to prevent overfitting by randomly omitting features during training. The final output sequence represents the 3D positions of the nine joints corresponding to the subsequent 100 time-normalized points.

**Figure 6:**
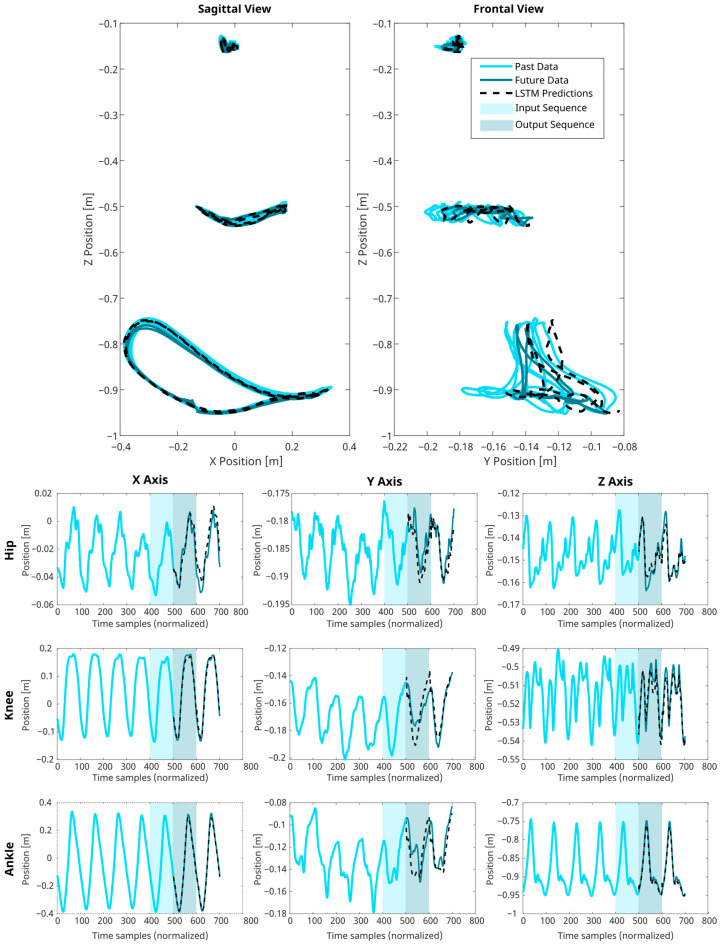
Results of LSTM Predictions for successive gait cycles. This figure illustrates the presented LSTM network’s capability to predict 3D joint trajectories. The known joint positions are delineated as light blue lines, LSTM’s predictions are represented as dashed black lines, and the true future positions are shown as teal lines. The input sequence depicting the last 100 time-normalized data points is determined by the shaded light blue areas. In contrast, the network’s prediction output for the given input is highlighted in the teal areas. The charts across the X-Z and Y-Z planes, alongside the temporal sequences for the X, Y, and Z axes, collectively affirm the model’s fidelity in replicating the intricate, rhythmic motions characteristic of human ambulation.

The model, as depicted in Figure 5, was designed to facilitate one-step-ahead predictions of gait trajectories. Its input layer is specifically formulated to receive sequences of 100 points (corresponding to a full gait cycle), each encompassing 11 distinct features. These input features represent the last known gait cycle data, including the subject’s height, gait velocity, and the position coordinates (x, y, z) of the hip, knee and ankle. Inside the model, two principal LSTM layers of 120 hidden units each and separated by two dropout layers (set at 0.2 to prevent overfitting) encode and decode the input data. The high-level features learned by the LSTMs are fed into a fully connected layer, translating them into sequences of 100 points of nine distinct output features corresponding to predictions of the 3D positions of the lower-extremity joints during the future gait cycle, see Figure 6.

#### 2.4.3. Model Training

**Figure 7:**
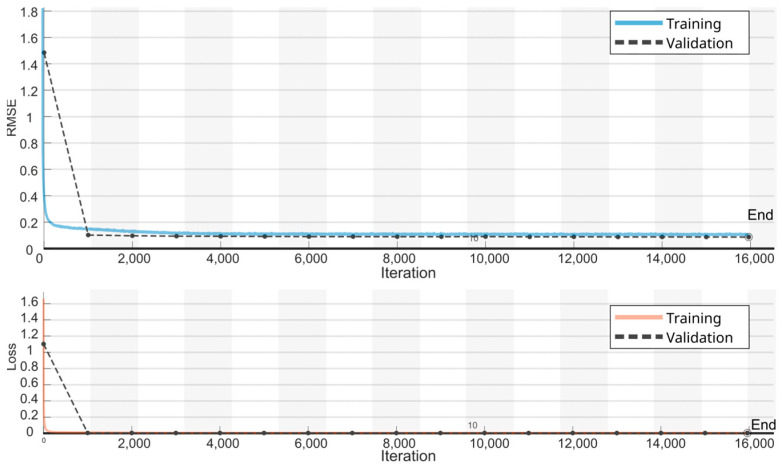
RMSE and Loss learning curves during the LSTM training. Solid lines represent the training set and dashed lines the validation set.

The LSTM model was trained on 60% of the walking sessions using the Adam optimizer [31]. The training involved 20 epochs, utilizing a mini-batch size of 256. The initial learning rate was set at 0.001 and adjusted dynamically according to a piecewise schedule every 125 epochs, reducing by a factor of 0.2. To mitigate the risk of exploding gradients, gradient clipping was employed with a threshold set at 1. To monitor and enhance model performance, validation checks were conducted every 1000 batches using 10% of the walking sessions designated as validation data, see Figure 7. Early stopping was implemented to halt training if no improvement in validation loss was observed for five consecutive epochs, thereby preventing overfitting.

### 2.5. Evaluation of Gait Models Accuracy

To evaluate the performance of the regression and LSTM models in estimating 3D gait trajectories of the ankle, knee, and hip, root mean squared error (RMSE) and Pearson’s correlation coefficient ($\rho_{\left(y,\hat{y}\right)}$) were used as evaluation metrics over the testing datasets (30% of the full dataset), defined as (8)–(9):

(8) $$RMSE=\sqrt{\frac{1}{N}\sum_{i=1}^{N}{\left(y_{i}-\hat{y_{i}}\right)}^{2}}$$

(9) $$\rho_{\left(y,\hat{y}\right)}=\frac{\sum_{i=1}^{n}\left(y_{a,i}-\bar{y_{a}}\right)\left(\hat{y_{b,i}}-\bar{\hat{y_{b}}}\right)}{\sqrt{\sum_{i=1}^{n}{\left(y_{a,i}-\bar{y_{a}}\right)}^{2}\sum_{j=1}^{n}{\left(\hat{y_{b,j}}-\bar{\hat{y_{b}}}\right)}^{2}}}=\frac{cov\left(y,\hat{y}\right)}{\sigma_{y}\sigma_{\hat{y}}}$$

The evaluation of the RMSE and correlations was conducted at various levels. The global model performance can be captured by the overall RMSE and correlation coefficient, indicated as $RMSE_{O}$ and $\rho_{O}$. At a more detailed level, the accuracy for each joint’s estimation in the X, Y and Z axes are presented by $RMSE_{J}$ and $\rho_{J}$. Variability across different walking trials was also examined, ${\mathrm{RMSE}}_{T}$ and $\rho_{T}$, to assess the model’s robustness to changes in trial-specific conditions (height and gait velocity). Additional step-level performance was analysed through $R=\{{\mathrm{RMSE}}_{i}|i=1,\ldots ,N\}$ and $C=\{\rho_{i}|i=1,\ldots ,N\}$, where N represents the number of steps per trial. 

## 3. Results

### 3.1. Regression-Based Model

All selected key-points exhibited similar dependencies for variations in height and gait speed, as presented in Table A1, Table A2 and Table A3. Of the 66 key-points, the key-point position parameters (*y)* showed statistically significant relationships (*p* < 0.05) with gait speed for 56 key-points, and with subject height for 54 key-points. For the time parameter (*t*), 38 key-points showed a significant relationship with gait speed, while 44 key-points were significantly related to height. These results indicate a comparable dependence on both factors, gait speed and body height on joint trajectories, which has not previously been reported in other angular generator models referenced in the literature [11]. The adjusted parameters from the robust regressions across all key-points for each joint axis are detailed in Table A1, Table A2 and Table A3. We report RMSE errors ranging from 0.01 m to 0.06 m for position (*y*) estimations and from 0.6% to 13.17% for time (*t*) estimations.

**Figure 8:**
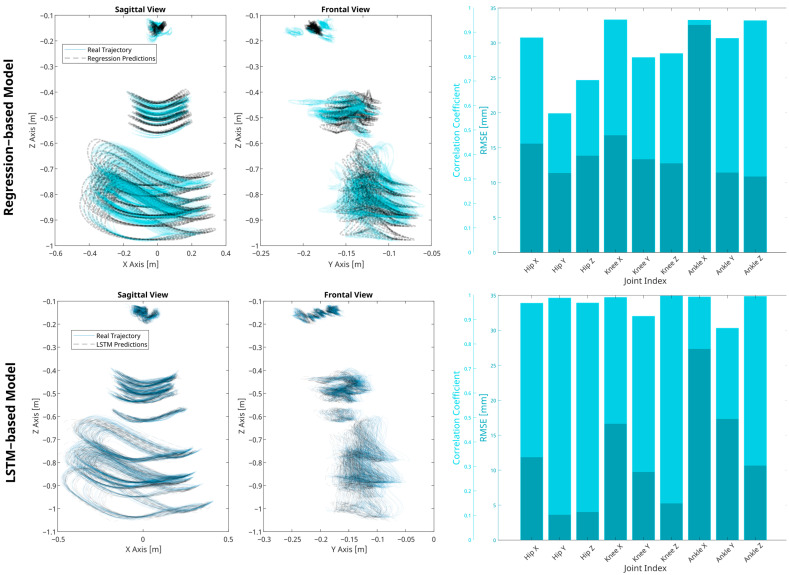
Validation of regression and LSTM-based models. Figures in the first row display the true gait trajectories of the training set in solid blue lines alongside the model predictions in dotted dashed black lines in both the sagittal and frontal planes, illustrating the models’ fidelity in spatial trajectory estimation. The second row contains bar graphs representing the correlation coefficients and RMSE metrics for each joint index, providing a quantitative assessment of each model’s performance.

**Figure A1:**
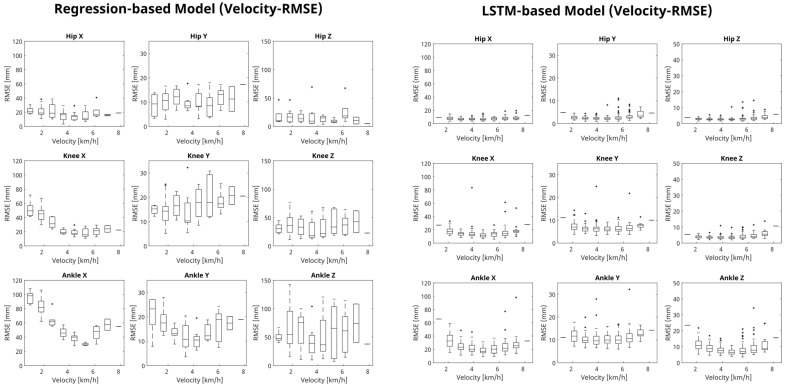
Comparative analysis of gait prediction error accuracy using regression-based and LSTM-based models under velocity variations. This figure displays box plots illustrating the RMSE (Root Mean Square Error) values for both regression-based and LSTM-based models across varying velocities (km/h) for the hip, knee, and ankle joints in the X, Y, and Z axes. Each box plot details the RMSE computed from true and estimated joint trajectories, highlighting the median, quartile ranges (Q1, Q3), and outliers. This visualization aids in evaluating the models’ precision in estimating gait movements under different walking speeds.

**Figure A2:**
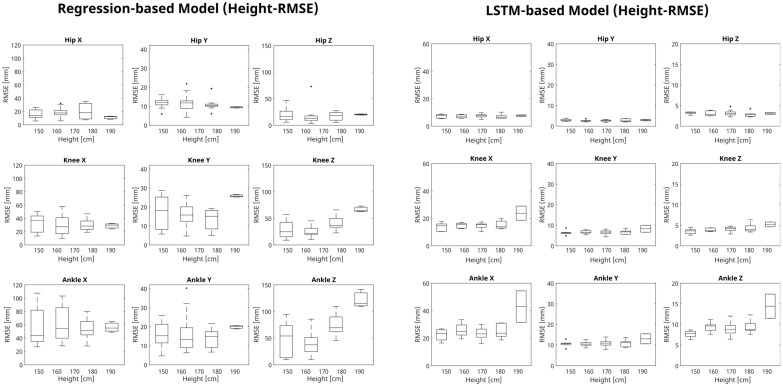
Comparative analysis of gait prediction error accuracy using regression-based and LSTM-based models under height variations. This figure displays box plots illustrating the RMSE (Root Mean Square Error) values for both regression-based and LSTM-based models across varying heights (cm) for the hip, knee, and ankle joints in the X, Y, and Z axes. Each box plot details the RMSE computed from true and estimated joint trajectories, highlighting the median, quartile ranges (Q1, Q3), and outliers. This visualization aids in evaluating the models’ precision in estimating gait movements under different subject heights.

**Figure A3:**
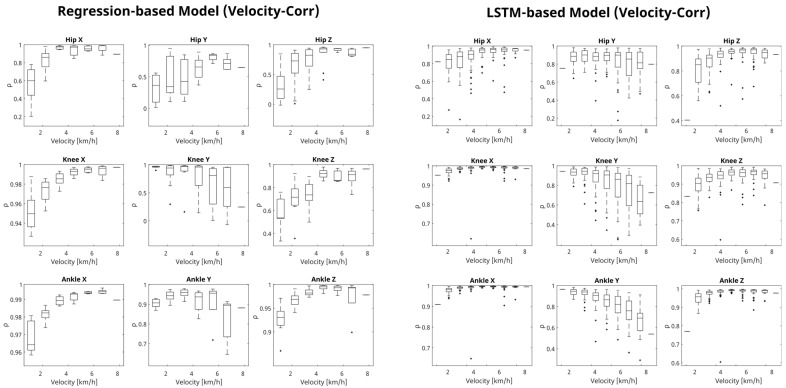
Comparative analysis of gait prediction correlation accuracy using regression-based and LSTM-based models under velocity variations. This figure displays box plots illustrating the correlation coefficient (ρ) values for both regression-based and LSTM-based models across varying velocities (km/h) for the hip, knee, and ankle joints in the X, Y, and Z axes. Each box plot details the correlation computed from true and estimated joint trajectories, highlighting the median, quartile ranges (Q1, Q3), and outliers. This visualization aids in evaluating the models’ precision in estimating gait movements under different walking speeds.

**Figure A4:**
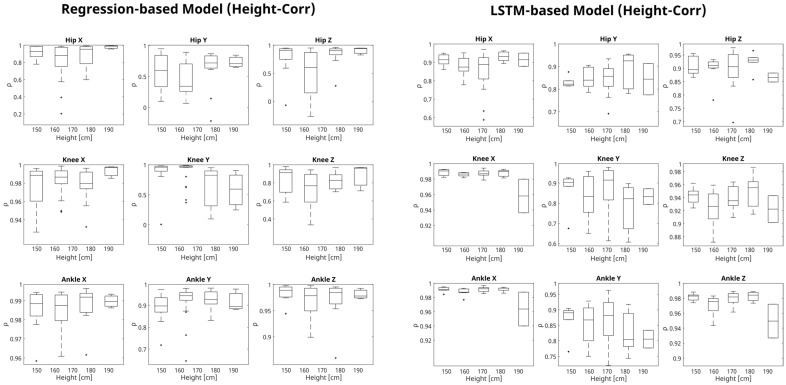
Comparative analysis of gait prediction correlation accuracy using regression-based and LSTM-based models under velocity variations. This figure displays box plots illustrating the correlation coefficient (*ρ*) values for both regression-based and LSTM-based models across varying heights (cm) for the hip, knee, and ankle joints in the X, Y, and Z axes. Each box plot details the correlation computed from true and estimated joint trajectories, highlighting the median, quartile ranges (Q1, Q3), and outliers. This visualization aids in evaluating the models’ precision in estimating gait movements under different subject heights.

Model accuracy was evaluated by comparing the reconstructed trajectories against the testing data. The overall root mean squared error ($RMSE_{O}$) was recorded at 13.40 mm, with an overall correlation coefficient ($\rho_{O}$) of 0.92, as highlighted in Table 2. At the joint level, detailed in Figure 8 and Table 2, the highest $RMSE_{J}$ value of 33.93 mm was recorded at the ankle-x axis, while the lowest error of 10.65 mm was observed at the hip-y axis. Despite the higher RMSE observed at the ankle joint, the strongest correlations, up to $\rho_{J}$ = 0.99, were also noted at the ankle (x, z axis) and also at the knee-x axis. This suggests that while the model effectively captures the directional trends of ankle movement, the large range of motion at this joint may amplify absolute errors, resulting in higher RMSE values. The weakest correlations were recorded at the hip y-axis with a value of $\rho_{J}$ = 0.71 primarily due to the nature of the variability of this joint and axis. Further analysis of the model robustness under varying trial-specific conditions revealed consistently strong performance, with mean ${\mathrm{RMSE}}_{T}$ values below 30 mm across all speeds and subject heights in the testing set. However, performance noticeably decreased near the boundary values of the dependent variables, as illustrated in Figure A1, Figure A2, Figure A3 and Figure A4.

### 3.2. LSTM-Based Model

Subsets of 100 points from the testing dataset served as inputs for the model performance evaluation. Comprehensive performance, characterized by the overall root mean squared error ($RMSE_{O}$) and Pearson correlation coefficient ($\rho_{O}$), yielded values of 12.57 mm and 0.99, respectively. Joint-specific evaluations were conducted for all axes, revealing ${RMSE}_{J}$ values below 27.39 mm, and correlation coefficients ($\rho_{J}$) above 0.95. When tested under varied walking conditions and subject characteristics, continued low RMSE errors with marginal error amplification at the boundaries of height and velocity parameters were observed, as can be observed in Figure A1, Figure A2, Figure A3 and Figure A4. Additionally, model performance at the step level was analysed through the computation of RMSE across all testing steps. As indicated in Table 3, the mean RMSE values ($\bar{R}$) and mean correlations ($\bar{C}$) for all steps at each joint axis were found to be similar to the $RMSE_{J}$ values, yet it is noted that the standard deviations at the step-level were far greater than those reported at the joint level.

## 4. Discussion

This study contributes to the evolving landscape of gait pattern generator models by integrating regression-based techniques and LSTM networks in order to predict three-dimensional gait patterns with high precision and adaptability. 

The presented regression model demonstrated strong predictive accuracy closely aligned with state-of-the-art angular gait generators when predicting joint trajectories of the lower limbs: Koopman et al. [9] reported an overall correlation coefficient for all joints of 0.91 (ours was 0.92); Hu et al. [12] reported $R^{2}$ values between 0.68 and 0.98 for the hip, knee and ankle (while those herein reported ranged from 0.5 to 0.98). An RMSE comparison with the aforementioned methods is not possible given a lack of consistency between reported outcomes and their normalization between studies. Regarding state-of-the-art three-dimensional gait pattern estimators: in our previous work [24], we obtained an overall RMSE of 25.73 mm compared to 13.4 mm obtained in the presented regression model; Di et al. [25] reported RSME for the ankle x, y and z axis of 59.1, 39.1 and 33.0 mm, respectively (ours were 33.93, 12.34 and 15.52 mm). 

The LSTM-based model defined in our study showcased superior adaptability to temporal variations, reflecting an overall correlation coefficient of 0.99 and $RMSE_{O}$ of 12.57 mm, with joint-specific $RMSE_{J}$ values ranging from 2.84 to 27.30 mm and correlation coefficients $\left(\rho_{J}\right)$ between 0.92–0.99. These results are similar to findings from previous studies; Luu et al. [14] reported correlation coefficients of 0.98 and 0.97 for predictions of angular trajectories of the hip and knee in the sagittal plane, respectively; Wu et al. [18] documented similar correlations with coefficients ranging from 0.97 to 0.99 again for the hip and knee movements in the sagittal plane. Despite a lower correlation coefficient of 0.92 reported at the ankle-y axis in our model, it is noted that this measure pertains to motion in the frontal plane, which was not evaluated in the aforementioned studies. Sagittal plane estimations (x and z axes for all joints) in our model ranged from 0.97 to 0.99, underscoring consistency with, and in some respects, enhancement over the results reported in prior research.

Evaluation of the model accuracies in capturing variability across different walking trials revealed that while both proposed methods demonstrated reliable performance for conditions within the mid-range spectrum of walking speeds and subject heights, the regression model exhibited unique limitations. Specifically, it exhibited reduced accuracy for trials at the dataset’s extremes of subject heights (147–192 cm) and gait velocities (1.29–8.02 km/h), as evidenced by lower correlations and higher errors, which are detailed in Figure A1, Figure A2, Figure A3 and Figure A4. In contrast, the LSTM model maintained consistent performance, with only slight precision decreases at these boundary conditions. It is noted that both models consistently underperformed in predicting trajectories in the y-axis in comparison to the x and z axes. This may be attributable to inherently greater variability in lateral movements during walking, which are often less constrained and more prone to person-specific patterns, hence presenting a more complex prediction task for both regression and LSTM methodologies.

When examining the predictive performance in estimating intra-trial variability, the LSTM model outperformed the regression model predictions as expected, as can be seen in Table 3. Although both models display a similar percentage of outliers, the LSTM model achieves lower mean RMSE ($\bar{R}$), standard deviations, and more narrow interquartile ranges, indicating less distribution of prediction errors. The LSTM model’s strengths can also be observed through the average correlations ($\bar{C}$) it achieved, which also exceeds that of the regression model. 

Although the differences in RMSE and correlation between models are noteworthy, it is essential to understand that each model has specific applications and offers solutions to a common problem from two different approaches. The regression model provides a robust solution in scenarios where data is limited or computational resources are constrained. Conversely, the LSTM model offers superior adaptability and accuracy in real-time dynamic environments. Together, both models enhance the overall effectiveness of gait rehabilitation systems by addressing different needs and constraints.

The value of the regression model becomes particularly evident in challenging scenarios for gait forecasting, such as with patients who have severely affected gait patterns and limited or unavailable gait data, or when computational resources are restricted. This model generates stable and reliable trajectories by relying solely on the user’s morphological values and desired speed, ensuring that end-effector gait rehabilitation robotic devices can effectively and safely guide the generated gait trajectories. Conversely, in situations where users are healthy or not severely affected and have available kinematic context data, the LSTM model excels. It adapts to real-time changes in gait dynamics, making it ideal for forecasting new trajectories and personalizing reference gait trajectories to finely tune to the evolving needs of the users. However, this comes at a greater computational cost.

This study has effectively demonstrated that both the presented regression model and the LSTM network are capable of estimating three-dimensional gait patterns at or above the level of current literature benchmarks in angular gait pattern generation [11,12,14,18]. These models provide precise estimations of gait variability across differing gait conditions, highlighting their potential utility for tailored gait rehabilitation and assistance. 

In summary, while both the regression and LSTM models demonstrate robust performance comparable to current state-of-the-art methods in gait analysis, they are inherently limited by the range of heights (147–192 cm), gait velocities (1.29–8.02 km/h), and ages included in the dataset. This limitation suggests that the models may not generalize well to gait patterns outside of these observed conditions. The regression model, chosen for its balance of performance, adaptability, and computational cost, provides an efficient solution under conditions of limited data or resources. In contrast, the LSTM model is favoured for its real-time adaptability, though it demands higher computational power. Addressing the disparity between these models, there is a clear opportunity to explore the potential of more advanced computational architectures. Such models could be designed to enhance adaptability and accuracy while maintaining a manageable computational footprint. This approach would not only strive for a balance between performance and computational efficiency but also ensure that these solutions fit within the hardware constraints currently prevalent in gait rehabilitation systems. 

Future work should focus on expanding the dataset to encompass a broader spectrum of subject characteristics, such as a wider range of ages and more diverse gait speeds. Additionally, integrating bilateral limb data could refine the estimation of inter-limb variability and balance, enhancing the comprehensiveness of gait analysis systems. Moreover, addressing the disparity between the current models suggests a clear opportunity to explore the potential of more advanced computational architectures. These should be carefully designed to enhance adaptability and accuracy while balancing the trade-off between computational efficiency and performance, fitting within the existing hardware constraints of gait rehabilitation systems. Beyond these constraints, pursuing precision without the limitations of current hardware, future research could also consider employing more computationally demanding models, such as larger and more complex Transformers. These models, known for their superior performance in tasks requiring contextual understanding, could lead to significant advancements in the precision and adaptability of gait analysis models [32].

## 5. Conclusions

This study introduces a dual approach for generating gait patterns, utilizing both regression-based and LSTM-based models to estimate three-dimensional gait trajectories. We aimed to extend beyond traditional gait pattern generators for robotic gait assistant purposes, which only focus on estimating angular parameters, to provide a comprehensive approach for three-dimensional joint gait pattern estimation. 

Both presented gait generators, regression-based and LSTM-based, achieved accuracies comparable to state-of-the-art models, offering precise estimations of gait variability across a range of gait speeds (1.29–8.02 km/h) and subject heights (147–192 cm). The presented regression-based model is particularly effective for estimating consistent predictions for specific gait conditions. It is ideally suited for gait rehabilitation in patients with significant physical impairment, where gait patterns may be unstable, severely affected, or virtually absent. Alternatively, the LSTM model excels at managing real-time, dynamic gait changes and is likely to be more beneficial in scenarios requiring advanced gait rehabilitation or assistance. In these settings, user gait trajectories are likely to be stable, and the task of the robotic system is therefore to adapt to inherent dynamic variability to enhance human–robot interaction. 

Both models demonstrate significant potential to enhance end-effector robotic devices by providing them with the capability to assist gait trajectories through a more personalized approach. This dual-model strategy not only offers a robust solution to the complexities of gait analysis but also enriches the potential for tailored therapeutic applications, significantly improving outcomes for a diverse range of patients.

## Figures and Tables

**Table 1:** Dataset description.

**Older Adults Cohort**					
**Variable**	**Median**	$P_{25}$	$P_{75}$	**Max**	**Min**
Height [cm]	164.5	151.5	168.2	175	147
Velocity [km/h]	4.4	3.3	5.4	7.6	1.3
Mass [kg]	70.25	64.7	73.1	79.7	46
Age	62	57	68	80	50
**Young Adults Cohort**					
**Variable**	**Median**	$P_{25}$	$P_{75}$	**Max**	**Min**
Height [cm]	171	166.8	180.6	192	153
Velocity [km/h]	4.6	3.3	5.6	8	1.4
Mass [kg]	66.3	61.2	77.6	95.4	44.9
Age	28	24	31	37	21

**Table 2:** Validation results when computing the joint specific (*J*) and overall (*O*) RMSE in millimetres and correlation for the validation set.

Joint	Axis	Regression Model				LSTM Model			
		$RMSE_{J}$	$RMSE_{O}$	$\rho_{J}$	$\rho_{O}$	$RMSE_{J}$	$RMSE_{O}$	$\rho_{J}$	$\rho_{O}$
Hip	X	15.62	13.40	0.92	0.92	8.09	12.57	0.96	0.99
	Y	10.65		0.71		2.84		0.98	
	Z	13.81		0.80		2.92		0.99	
Knee	X	19.94		0.99		16.20		0.99	
	Y	14.03		0.80		6.92		0.95	
	Z	14.83		0.90		4.75		0.99	
Ankle	X	33.93		0.99		27.39		0.99	
	Y	12.34		0.92		11.31		0.92	
	Z	15.52		0.99		10.86		0.99	

**Table 3:** RMSE and Pearson’s correlation coefficient variability for each joint axis estimation. RMSE and correlation coefficients were computed for each estimated step, ${\mathrm{RMSE}}_{step}\;\mathrm{and}\;\rho_{step}$ respectively, for all axes of each joint. The average, standard deviations, quartiles and percentage of outliers are computed for each R and C to illustrate the model accuracies for each step.

Model	Joint	Axis	R					C				
			$\bar{R}$	σ	Q1	Q3	$O_{\%}$	$\bar{C}$	σ	Q1	Q3	$O_{\%}$
Regression	Hip	X	19.63	11.15	11.77	24.28	5.73	0.85	0.24	0.85	0.97	9.63
		Y	12.16	8.01	6.54	16.05	3.90	0.66	0.31	0.51	0.89	3.38
		Z	15.71	13.80	7.18	20.25	4.15	0.76	0.21	0.69	0.91	5.22
	Knee	X	26.76	13.74	18.95	31.10	4.28	0.98	0.06	0.98	0.99	8.31
		Y	15.35	8.19	9.19	19.34	3.28	0.74	0.36	0.71	0.96	12.11
		Z	14.89	9.07	7.75	21.46	1.13	0.85	0.13	0.81	0.93	4.12
	Ankle	X	46.96	22.36	36.33	51.33	5.54	0.98	0.05	0.98	0.99	8.79
		Y	15.85	7.03	10.61	19.76	2.09	0.85	0.16	0.81	0.95	7.76
		Z	17.30	10.05	10.18	21.29	5.09	0.97	0.07	0.97	0.99	7.73
LSTM	Hip	X	6.79	4.00	4.47	8.14	3.91	0.94	0.12	0.94	0.99	11.05
		Y	2.43	1.93	1.55	2.82	4.28	0.92	0.13	0.91	0.98	8.61
		Z	2.81	1.94	1.96	3.24	3.98	0.95	0.09	0.95	0.99	10.42
	Knee	X	13.40	9.45	8.45	15.91	4.20	0.99	0.05	0.99	1.00	9.95
		Y	6.17	3.39	4.08	7.44	3.49	0.89	0.17	0.88	0.98	10.66
		Z	3.74	2.47	2.54	4.31	4.57	0.96	0.07	0.96	0.99	8.10
	Ankle	X	20.97	18.14	11.85	24.84	5.19	0.99	0.05	1.00	1.00	11.54
		Y	9.93	5.38	6.41	12.22	3.37	0.91	0.14	0.89	0.98	8.98
		Z	7.93	6.35	4.67	9.29	5.27	0.98	0.06	0.99	1.00	11.48

## Data Availability

The processed data and results generated during the study are not publicly available but can be made available by the authors upon request. The raw data used in the study is available in Fukuchi, C.A.; Fukuchi, R.K.; Duarte, M. A Public Dataset of Overground and Treadmill Walking Kinematics and Kinetics in Healthy Individuals. PeerJ 2018, 6, e4640. https://doi.org/10.7717/PEERJ.4640 (accessed on 9 May 2024).

## Appendix A

**Table A1:** Regression results for the hip key values in the x, y and z axis.

Hip (x,y,z)								
Axis	Parameter	Key-Point	Charact.	*β*_0_ (*Intercept*)	*β*_1_ (*Speed*)	*β*_2_ (*Speed^2^*)	*β*_3_ (*Height*)	*RMSE* [mm]
X	*t* *(% gait)*	1	Heel contact	1.00	-	-	-	-
		2	Max. y.	7.61	5.46	−1.86	−4.90	1.75
		3	Min. dy/dt	21.02	−7.41	2.36	−0.46	3.31
		4	Min. y.	64.37	−20.42	6.66	6.08	6.78
		5	Max. dy/dt	71.10	0.64	−1.84	7.09	5.11
		6	End of the gait cycle	100	-	-	-	-
	*y* *(position [m])*	1	Heel contact	0.03	0.03	-	−0.04	20.33
		2	Max. y.	0.02	0.03	−0.01	−0.02	20.42
		3	Min. dy/dt	0.02	0.01	-	−0.02	19.51
		4	Min. y.	-	0.02	−0.02	−0.02	17.66
		5	Max. dy/dt	−0.02	0.04	−0.02	-	17.15
		6	End of the gait cycle	0.03	0.03	0	−0.04	20.31
Y	*t* *(% gait)*	1	Heel contact	1.00	-	-	-	-
		2	Min. y (early stance)	8.84	6.50	−1.47	−2.41	3.90
		3	Min. dy/dt	24.53	−5.35	3.50	1.20	3.50
		4	Max. y.	37.50	−15.19	7.75	5.52	5.19
		5	Min. pos (late stance)	58.89	−5.47	3.19	−5.74	3.32
		6	Min. dy/dt	78.90	3.73	−1.10	−15.52	4.05
		7	Min. y.	95.94	−7.92	3.56	−2.14	4.03
		8	End of the gait cycle	100	-	-	-	-
	*y* *(position [m])*	1	Heel contact	−0.17	0.01	-	−0.03	13.03
		2	Min. y (stance)	−0.17	-	-	−0.02	13.52
		3	Min. dy/dt	−0.16	-	-	−0.02	13.15
		4	Max. y.	−0.16	−0.01	-	−0.02	13.02
		5	Min. pos (stance)	−0.17	−0.01	0.01	-	14.34
		6	Min. dy/dt	−0.18	0.01	-	−0.02	14.45
		7	Min. y.	−0.18	0.01	-	−0.02	14.64
		8	End of the gait cycle	−0.17	0.01	0	−0.03	13.03
Z	*t* *(% gait)*	1	Heel contact	1.00	-	-	-	-
		2	Max. y	11.72	−3.63	1.10	3.10	4.20
		3	Min. y.	34.72	−0.22	−0.41	−1.06	3.26
		4	Max. dy/dt (stance)	41.51	−5.41	2.32	1.48	2.56
		5	Min. dy/dt	54.43	0.72	−1.20	−2.40	2.31
		6	Min. y.	72.00	−12.51	4.99	4.05	3.17
		7	Max. dy/dt	80.73	−5.01	2.29	0.62	2.33
		8	Max. y.	91.27	−1.41	1.18	−3.13	3.28
		9	End of the gait cycle	100	-	-	-	-
	*y* *(position [m])*	1	Heel contact	−0.06	−0.01	-	−0.10	19.23
		2	Max. y	−0.05	0.01	-	−0.12	19.61
		3	Min. y.	−0.07	0.01	-	−0.11	108.53
		4	Max. dy/dt (stance)	−0.05	-	-	−0.13	18.92
		5	Min. dy/dt	−0.05	0.01	-	−0.13	18.35
		6	Min. y.	−0.05	−0.02	-	−0.12	19.07
		7	Max. dy/dt	−0.05	−0.01	-	−0.12	19.4
		8	Max. y.	−0.05	−0.01	-	−0.12	19
		9	End of the gait cycle	−0.06	−0.01	-	−0.10	19.25

**Table A2:** Regression results for the knee key values in the x, y and z axis.

Knee (x,y,z)								
Axis	Parameter	Key-Point	Charact.	*β*_0_ (*Intercept*)	*β*_1_ (*Speed*)	*β*_2_ (*Speed^2^*)	*β*_3_ (*Height*)	*RMSE* [mm]
X	*t* *(% gait)*	1	Heel contact	1.00	-	-	-	-
		2	Max. dy/dt	3.00	-	-	-	2.82
		3	Min. dy/dt	5.20	−9.52	6.33	−14.31	5.34
		4	Min. y	29.98	−2.57	0.71	0.93	1.55
		5	Max. dy/dt	59.08	−7.07	1.81	−0.46	1.67
		6	Max. ye	75.94	−6.09	1.26	−4.99	1.99
		7	End of the gait cycle	100	-	-	-	-
	*y* *(position [m])*	1	Heel contact	−0.02	0.09	−0.01	0.11	21.90
		2	Max. dy/dt	−0.03	0.10	−0.02	0.12	21.99
		3	Min. dy/dt	-	0.08	−0.01	0.12	18.78
		4	Min. y	−0.08	0.04	−0.02	0.08	21.54
		5	Max. dy/dt	−0.09	−0.03	−0.01	0.03	20.5
		6	Max. ye	-	0.03	−0.03	0.01	26.16
		7	End of the gait cycle	−0.01	0.09	−0.01	0.11	21.85
Y	*t* *(% gait)*	1	Heel contact	1.00	-	-	-	-
		2	Max. y.	30.02	−1.11	0.28	8.23	10.50
		3	Min. dy/dt	41.73	14.48	−7.91	37.34	13.17
		4	Min. y.	58.46	−6.93	−0.22	10.27	5.12
		5	End of the gait cycle	100	-	-	-	-
	*y* *(position [m])*	1	Heel contact	−0.11	0.03	−0.01	−0.07	10.78
		2	Max. y.	−0.08	−0.02	0.01	−0.03	10.16
		3	Min. dy/dt	−0.15	0.03	−0.01	−0.06	16.89
		4	Min. y.	−0.12	0.01	-	−0.04	21.23
		5	End of the gait cycle	−0.11	0.03	−0.01	−0.07	10.89
Z	*t* *(% gait)*	1	Heel contact	1.00	-	-	-	-
		2	Max. dy/dt (stance)	2.86	3.15	−0.77	−1.96	1.74
		3	Max. y.	6.28	5.74	−1.27	−4.89	3.46
		4	Min. dy/dt	18.29	−0.80	0.32	1.72	1.97
		5	Min. y.	27.54	−5.80	1.44	9.67	2.56
		6	Max. y. (stance)	57.28	−1.98	−0.15	−1.37	1.57
		7	Min. dy/dt	68.39	−3.10	0.60	−6.33	1.80
		8	Min. y	72.50	−5.80	1.93	−3.10	2.04
		9	Max. y	88.92	−1.24	1.42	−3.51	3.8
		10	End of the gait cycle	100	-	-	-	-
	*y* *(position [m])*	1	Heel contact	−0.02	0.01	-	−0.57	14.23
		2	Max. dy/dt (stance)	−0.02	0.01	-	−0.57	14.99
		3	Max. y.	−0.04	0.02	-	−0.57	47.79
		4	Min. dy/dt	−0.03	0.02	-	−0.57	15.04
		5	Min. y.	−0.03	0.02	-	−0.56	21.74
		6	Max. y. (stance)	−0.02	0.01	0	−0.59	18.36
		7	Min. dy/dt	-	−0.01	-	−0.61	18.35
		8	Min. y	−0.02	-	-	−0.59	18.09
		9	Max. y	0.01	0.02	0	−0.63	61.67
		10	End of the gait cycle	-	−0.01	0.01	−0.60	14.24

**Table A3:** Regression results for the ankle key values in the x, y and z axis.

Ankle (x,y,z)								
Axis	Parameter	Key-Point	Charact.	*β*_0_ (*Intercept*)	*β*_1_ (*Speed*)	*β*_2_ (*Speed^2^*)	*β*_3_ (*Height*)	*RMSE* [mm]
X	*t* *(% gait)*	1	Heel contact	1.00	-	-	-	-
		2	Middle point	33.37	−5.87	1.48	1.32	0.71
		3	Min. y.	65.98	−11.19	2.77	2.53	1.31
		4	Max. dy/dt	77.19	9.27	−3.17	3.26	1.82
		5	Max. y.	93.19	2.67	−0.82	1.92	0.88
		6	End of the gait cycle	100	-	-	-	-
	*y* *(position [m])*	1	Heel contact	−0.03	0.20	−0.04	0.14	30.98
		2	Middle of k1–k3	−0.01	0.04	−0.03	−0.07	21.81
		3	Min. y.	-	−0.12	-	−0.25	29
		4	Max. dy/dt	−0.17	0.28	−0.09	0.08	31.44
		5	Max. y.	-	0.19	−0.04	0.14	34.52
		6	End of the gait cycle	−0.03	0.2	−0.05	0.14	31.59
Y	*t* *(% gait)*	1	Heel contact	1.00	-	-	-	-
		2	Max. dy/dt	12.28	0.64	0.15	6.50	1.92
		3	Max. y.	24.33	1.09	0.41	12.84	3.87
		4	Max. dy/dt	66.48	−5.59	−0.23	7.31	4.35
		5	Max. y	82.66	−1.33	−0.46	−0.26	2.21
		6	Min. dy/dt	93.81	4.64	−1.34	−3.77	1.70
		7	End of the gait cycle	100	-	-	-	-
	*y* *(position [m])*	1	Heel contact	−0.13	0.05	−0.01	−0.03	11.03
		2	Max. dy/dt	−0.07	−0.01	-	−0.01	9.66
		3	Max. y.	−0.05	−0.04	0.01	-	10.57
		4	Max. dy/dt	−0.16	0.04	−0.01	-	15.17
		5	Max. y	−0.14	0.06	−0.01	−0.04	15.94
		6	Min. dy/dt	−0.14	0.05	−0.01	−0.04	13.69
		7	End of the gait cycle	−0.13	0.05	−0.01	−0.04	11.22
Z	*t* *(% gait)*	1	Heel contact	1.00	-	-	-	-
		2	Min. dy/dt	15.05	−0.49	0.14	−0.29	1.68
		3	Min. y.	29.12	−1.10	0.34	0.09	3.34
		4	Max. dy/dt	68.28	−4.31	−1.21	0.38	2.89
		5	Max. y.	70.65	−5.04	0.15	4.48	1.51
		6	Min. dy/dt	72.31	4.54	−2.87	7.29	1.89
		7	Min. y.	90.68	−2.00	0.17	1.18	0.60
		8	End of the gait cycle	100	-	-	-	-
	*y* *(position [m])*	1	Heel contact	-	0.03	−0.01	−1.07	16.36
		2	Min. dy/dt	−0.01	0.02	-	−1.07	16.4
		3	Min. y.	−0.02	-	-	−1.05	15.95
		4	Max. dy/dt	−0.03	0.12	−0.03	−0.99	23.08
		5	Max. y.	−0.06	0.11	−0.02	−0.92	17.93
		6	Min. dy/dt	−0.01	0.03	-	−1.00	17.36
		7	Min. y.	-	0.01	-	−1.05	15.66
		8	End of the gait cycle	0	0.03	−0.01	−1.07	16.42
